# Minimally Invasive Intra-Articular Anterior Cruciate Ligament Reconstruction With Additional Extra-Articular Tenodesis Using Autologous Hamstrings With Over-the-Top Technique

**DOI:** 10.1016/j.eats.2025.103567

**Published:** 2025-05-14

**Authors:** Guo Yang, Song Hongyu

**Affiliations:** Department of Orthopedics and Sport medicine, The First Affiliated Hospital of Xiamen University, Xiamen, China

## Abstract

We describe a minimally invasive surgical method to perform integrated reconstruction of intra-articular and extra-articular ligaments using the hamstring tendon as the graft. The process of intra-articular anterior cruciate ligament reconstruction and femoral over-the-top technique is completed under the arthroscope, and the reconstruction of the anterior lateral ligament outside the joint is completed using the percutaneous technique. Its simplicity and convenience enable us to apply in anterior cruciate ligament revision surgery.

Numerous surgical methods have been introduced to stabilize the knee joint in cases of anterior cruciate ligament (ACL) rupture. Anterior cruciate ligament reconstruction (ACLR) is aimed at restoring knee joint stability, facilitating activity recovery, and preventing secondary damage to the cartilage and meniscus.^1^ For patients with ACL injuries who have positive result after pivot shift test examination, traditional ACLR cannot reliably restore the normal biomechanical stability of tibial rotational movement and requires enhancement of the lateral ligaments.^2^ The over-the-top (OTT) technique involves passing the graft through the superomedial edge of the lateral femoral condyle and fixing it on the lateral femoral cortex.^3^ On the basis of the OTT technique, we present a minimally invasive surgical method to perform integrated reconstruction of intra-articular and extra-articular ligaments using the hamstring tendon as the graft. We also provide a video showing the intra-articular OTT reconstruction process (Fig 1, Video 1).

**Fig 1:**
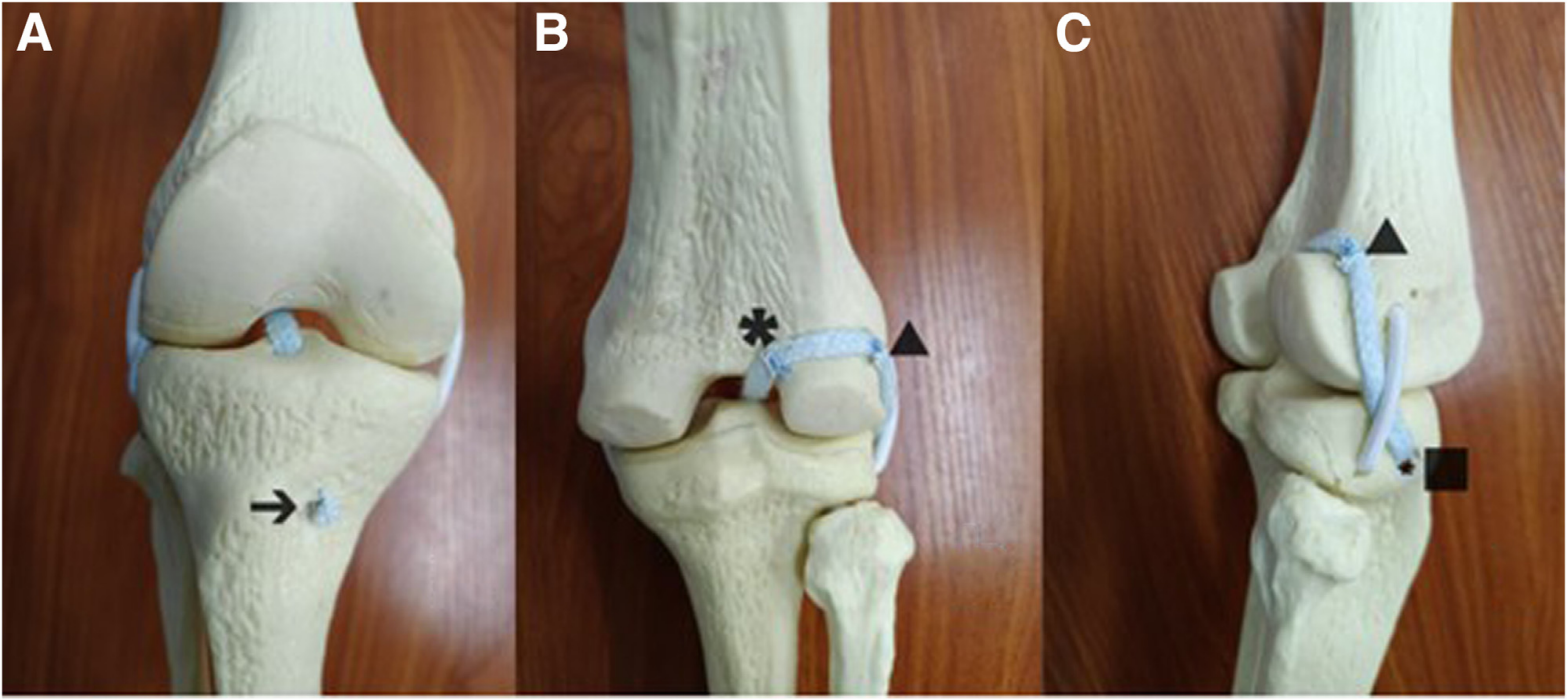
Overview of over-the-top + anterior lateral ligament integrated reconstruction: anterior view (A); posterior view (B); lateral view (C). The arrow shows the medial femoral bone tunnel of the tibia; ∗the internal anchor; ▲ the outer anchor; ▪ the extra-articular lateral bone tunnel of the tibial.

## Surgical Technique

The patient is positioned supine on the operating table. A pneumatic tourniquet is placed at upper middle part of the thigh. During the entire surgical procedure, it is essential to ensure a completely unrestricted range of motion of the knee joint. The knee should be kept bent at 90° without any artificial assistance, and proper support for the foot must be ensured. The body projections, including anterior tibial tuberosity, tibiofemoral joint line, fibular head, Gerdy tubercle, hamstring tendon position, and patellar tendon boundary, are identified. Conventional anterior medial and anterior lateral arthroscopic approaches are established. Accurate arthroscopic diagnosis is performed to assess injuries of the cruciate ligaments, menisci, and articular cartilage. Any accompanying articular cartilage or meniscus injuries are treated under the arthroscope. We make every effort to protect the meniscus tissue and perform meniscectomy and suture according to the different locations and degrees of the injury. Unstable articular cartilage defects are scraped off, and a spiked osteotome is used to freshen the cartilage injury.

The entire intercondylar notch is then examined for the presence of bony hyperplasia. In cases in which there are large osteophytes on the medial edge of the lateral condyle, we perform enlargement and shaping of the narrowed intercondylar fossa to prevent possible impingement on the ACL.

**Fig 2:**
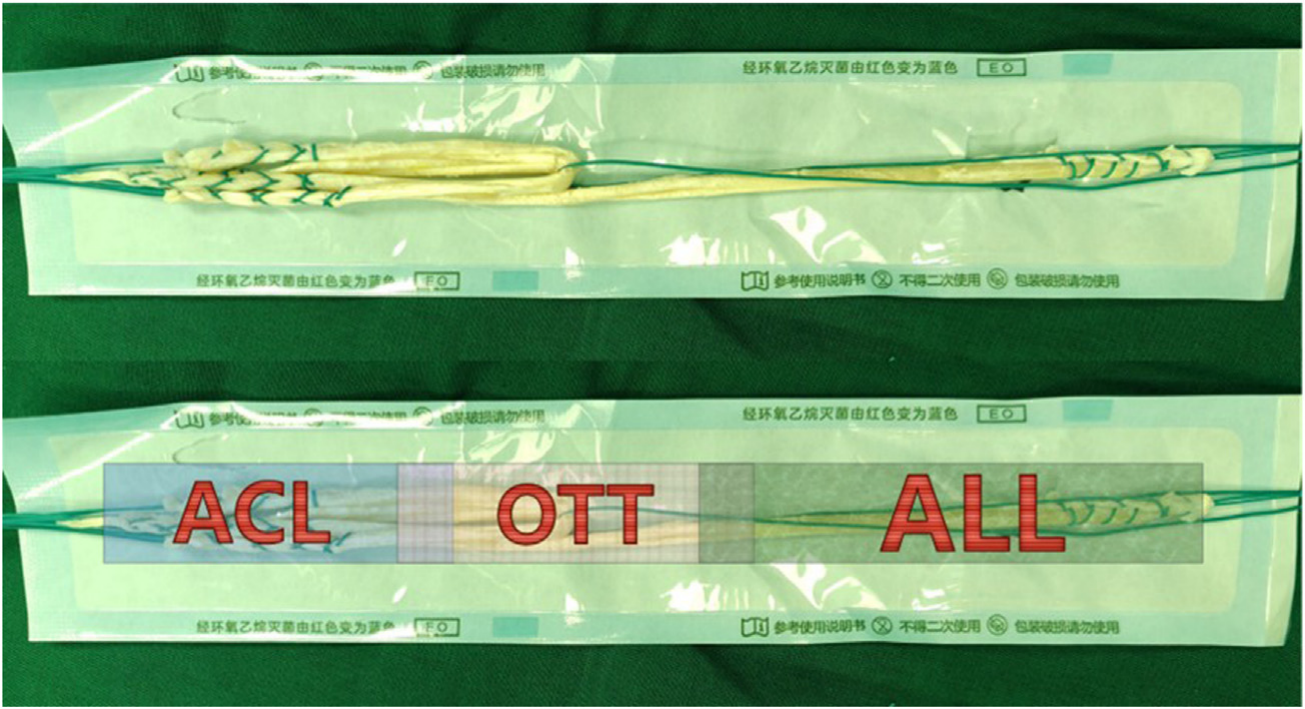
The autologous tendon obtained is braided into the graft. The total length exceeds 18 cm. Ensure that the length of each segment of the graft is at least as follows: anterior cruciate ligament (ACL) 5-7 cm, over-the-top (OTT) 4-5 cm, anterior lateral ligament (ALL) 6-8 cm.

Once ACL rupture is confirmed under arthroscopy, the gracilis and semitendinosus tendons are obtained. The removed tendons are sutured in a carpet-like manner with nonabsorbable thread to ensure that the total length of the graft is more than 18 cm and the diameter is not less than 7 mm (Fig 2). Part of the peroneus longus tendon is recruited as a supplementary source of the graft. The suture threads at the free ends of the tendons need to be tightened to obtain sufficient traction force, thus making it easier for the graft to pass through the bony tunnel.

**Fig 3:**
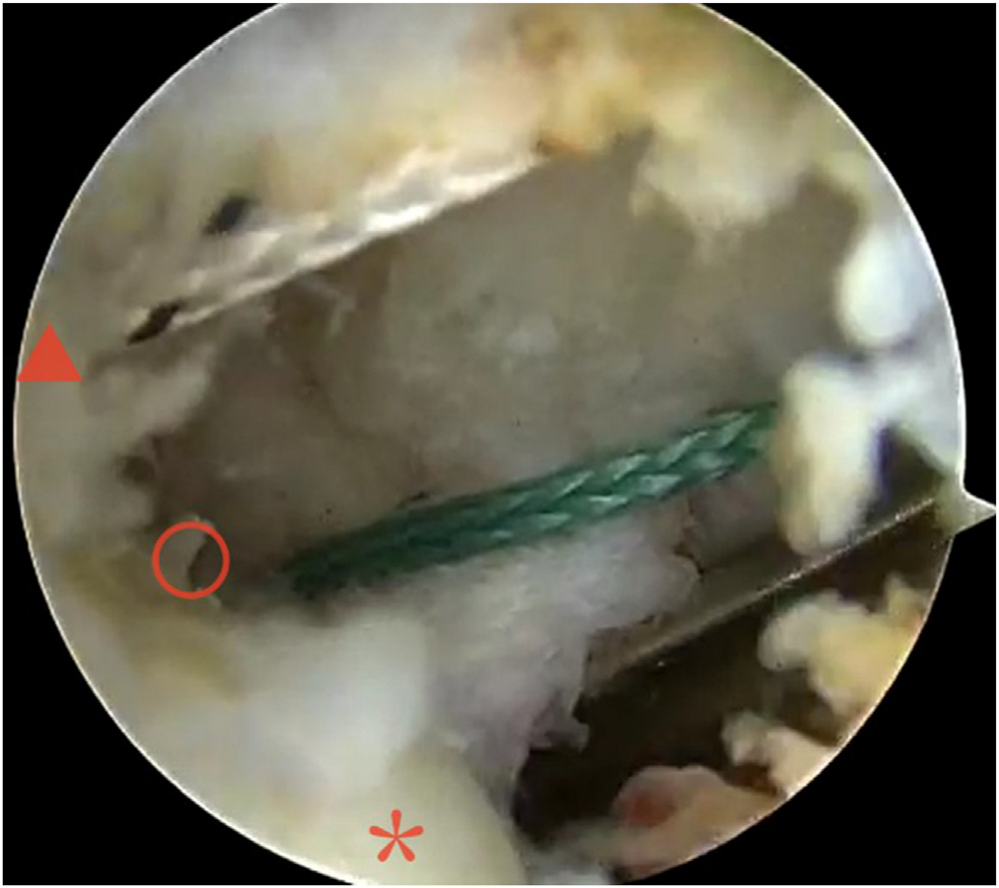
Imaging of internal anchor implantation under arthroscopy.The arthroscopy was placed via medial approach to observe the medial side of the lateral femoral condyle of the patient's right knee joint.⭐ the lateral medial surface of the femoral epicondyle; ⚪ the internal anchor;▲ the Entrance to the over-the-top section.

**Fig 4:**
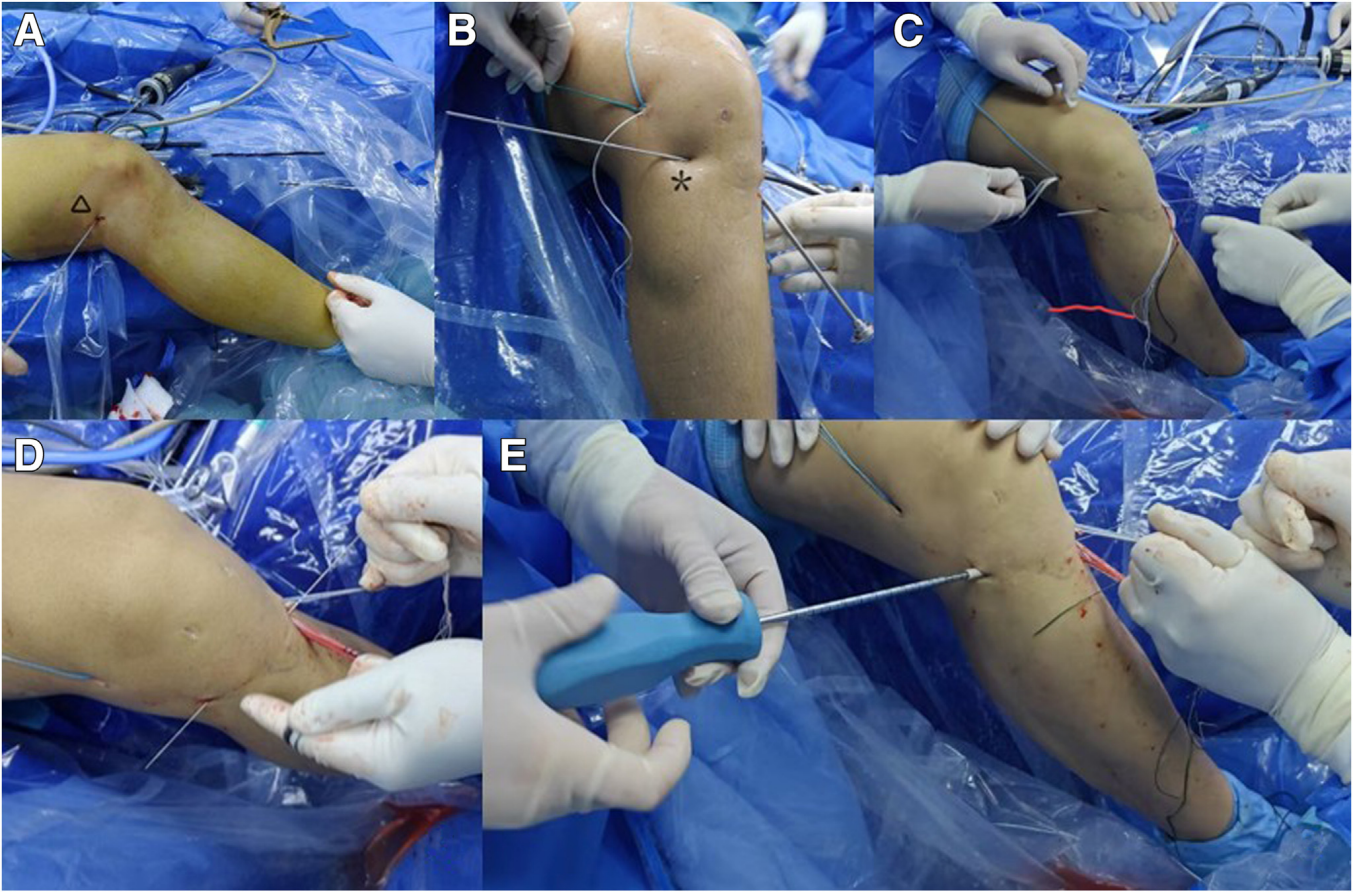
Surgical steps outside the arthroscope. (A) The right knee joint can be visualized from the lateral side. The skin incision and subcutaneous preparation at the lateral femoral epicondyle (▲) have been completed. The fascia is longitudinally opened, and a suture anchor has been placed at the lateral femoral epicondyle. (B) An incision (∗) is made below the level of the articular surface between the Gerdy tubercle and the fibula on the lateral side of the tibia. A 4.5-mm drill bit is used to drill a hole into the medial side of the tibia, creating an extracapsular tunnel. (C) The graft is introduced from the lateral side of the femur through a deep fascial soft tissue tunnel into the tibial bone tunnel using a traction suture. (D) The graft is then tensioned. (E) A compression screw is used to secure the graft at the lateral tibial entry point.

The medial surface of the lateral femoral condyle is exposed behind the posterior cruciate ligament. Under the arthroscopic monitoring, a curved bone rasp is used through the posterior medial approach to prepare a soft-tissue tunnel for the passage of the graft at the OTT position of the posterior femoral condyle, between the posterior wall of the lateral femoral condyle and the joint capsule. Then, a full-suture anchor (PEEK [polyether ether ketone] Corkscrew FT 4.5 ×14 mm; Arthrex) is placed at the highest point of the posterior edge of the medial surface of the lateral femoral condyle as an internal anchor point for standby (Fig 3). A 2-cm incision is made at the lateral femoral condyle. The skin, subcutaneous tissue, and fascia lata are successively incised until the femur is reached and the highest point at the posterior margin of the lateral femoral condyle is exposed. Another full-suture anchor (PEEK Corkscrew FT 4.5 ×14 mm) also is placed as an external anchor point. The curved wire passer is passed around the posterior condyle through the soft-tissue tunnel and emerges from the lateral femoral incision with a wire. The arthroscope is inserted via the posterior medial approach to locate the previously implanted internal anchor nail on the medial surface of the lateral femoral condyle. Grasping forceps are used to pass through different-colored internal anchor threads via the anterior medial approach to grasp one end of the wire and lead it out. The other end of the wire is retained outside the lateral condyle incision of the femur and the guidewire is introduced. The tibial-side bone tunnel is prepared with reference to 3-mm anterior and medial to the original anterior cruciate ligament insertion site with an Arthrex FlipCutter guide. Under arthroscopic monitoring, the guidewire is passed through the tibial tunnel using the wire gripper. Under arthroscopic surveillance, use grasping forceps to pass the guidewire through the tibial tunnel. The prepared graft is introduced from the tibial side and led out from the lateral femoral side through the guidewire, leaving the corresponding length of the graft outside the joint for strengthening the anterior lateral bundle ligament outside the joint. A 1-cm incision is made between the Gerdy tubercle and the fibular head below the joint surface, pointing obliquely medially to prepare an extra-articular bone tunnel at the tibial end. The traction wire is placed through the extra-articular bone tunnel, the deep layer of the lateral collateral ligament to the lateral femoral incision. The reserved anterior lateral bundle ligament is passed through the anterior medial tibial bone tunnel. The graft is tightened and the knee joint is flexed and extended multiple times. The internal anchor nail is tightened and tied, the external anchor nail is tied, and the tibial medial and lateral compression screw is fixed in a counterclockwise fashion. A toothed gate-shaped nail in the anterior medial tibial incision is used to fix the ligament (Fig 4 and Table 1).

**Table 1:** Steps, Pearls, and Pitfalls

Steps	Pearls	Pitfalls
1. Obtaining tendon.	Ensure the tendon has sufficient diameter and length.	Risk of inadequate healing because of insufficient diameter or length.
2. In the posteromedial approach, the surgeon exposes the lateral femoral condyle and inserts an internal anchor under arthroscope.	The internal anchor is positioned on the extension of the anterior cruciate ligament femoral anatomical point, mimicking the physiological course of the ligament.	If the inner anchor is too low, there is a possibility that the over-the-top ligament will slip.
3. The external anchor is placed at the lateral femoral condyle transitioned to the femoral shaft.	Limited incision of the fascia lata helps reduce the occurrence of lateral thigh pain after surgery.	Before percutaneous implantation of the external anchor, the soft tissue needs to be passively separated deeply as the bone surface.
4. Creation of the extra-articular lateral tibial bone tunnel.	The use of a sight avoids interaction with the medial tibial bone tunnel.	Excessively high, posterior extra-articular tracts may result in immobilization failure or nerve damage.
5. Arthroscopic preparation of the medial femoral tract of the tibia.	The diameter of the bone tunnel is selected according to the actual condition of the graft.	Bone canal integrity is important for revision surgery.
6. Insertion and fixation of the graft ligament.	The head and tail ends of the graft are knotted on the medial side of the tibia to form a closed loop.	Ligament tension needs to be rechecked after fixation; a loose graft cannot provide joint stability.

## Discussion

The history of ACLR is long and its method has been continuously developing. After a hundred years of development, ACLR surgery is considered to be approaching perfection, but there are still reported revision rates.^4^^,^^5^ High-risk patients (those who are young, active in pivoting sports, have a high-grade pivot-shift, generalized ligamentous laxity or knee hyperextension, Segond fracture, chronic ACL lesion, lateral femoral notch sign, lateral coronal plane laxity, concurrent meniscus repair, or anterolateral complex injury on magnetic resonance imaging) and modifiable risk factors (graft choice, graft size, tunnel position, graft fixation, associated injuries such as a lateral meniscal root tear, or anatomic factors such as an increased posterior tibial slope) should not be ignored.^6^ The combination of extra-articular and intra-articular reconstruction is considered necessary protect the graft from excessive and unwanted pressure in the early postoperative period; therefore, it is useful in ACL revision surgery. The combination of OTT and anterior lateral tendon fixation can better control the situation of rotational instability of the tibia, and its effect in controlling static knee joint laxity is similar to that of anatomic double-bundle reconstruction.^7^^,^^8^ Patients who underwent revision ACLR with a laterally based augmentation procedure had a lower failure rate than patients who underwent isolated revision ACLR. KT-1000 and pivot-shift examination were also significantly better when a lateral augmentation was performed.^9^ OTT-ACL all-around (anterior lateral ligament [ALL]) reconstructive technique shows a greater rate of success compared with traditional methods^10^^,^^11^ and provides more structural stability.^11^

**Fig 5:**
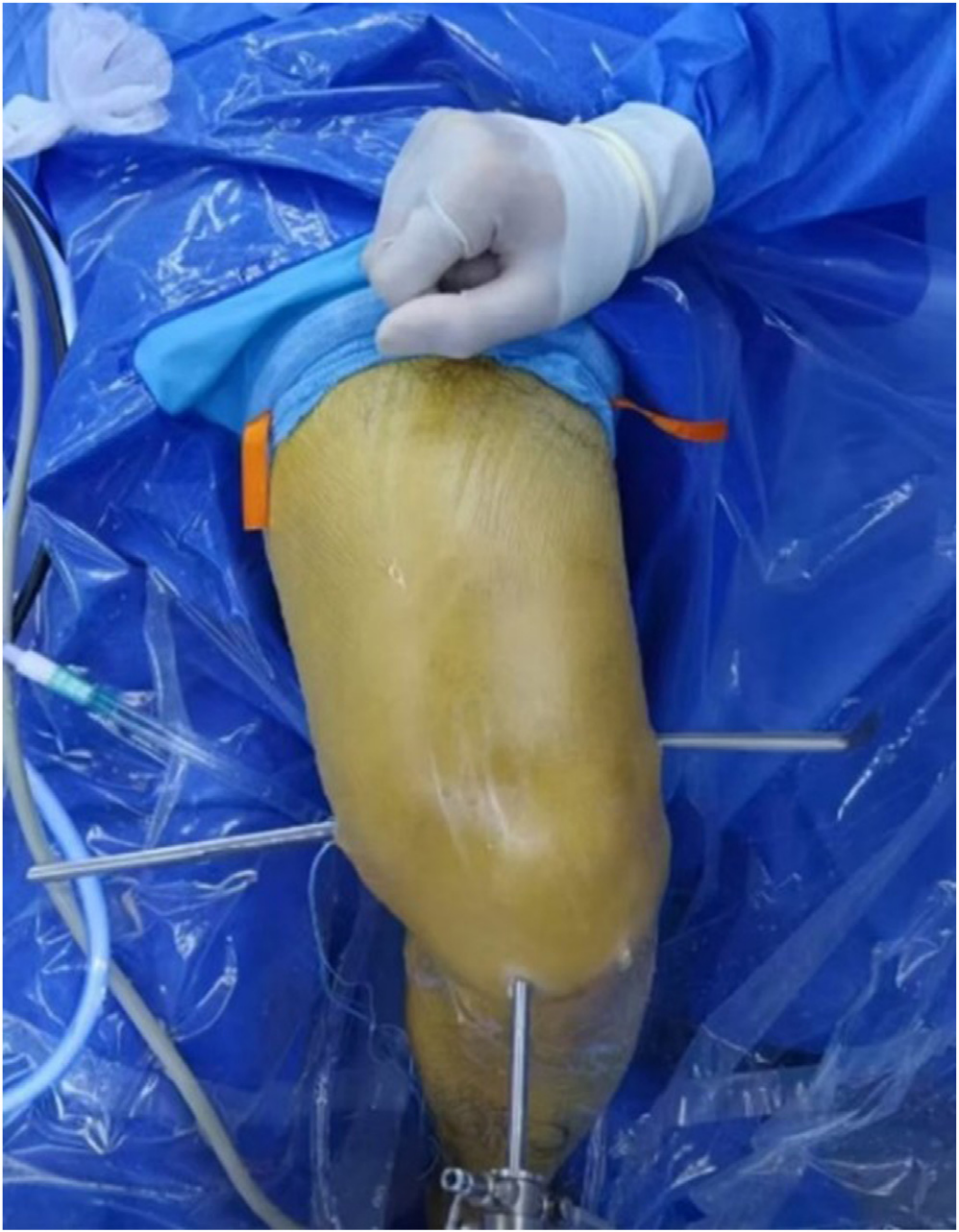
The surgical incisions include anterior medial, anterior lateral, and posterior medial arthroscopic approaches; the incision for the implantation of the lateral femoral external anchor, and the incision for the lateral tibial bone tunnel and the incision for obtaining the medial tendon.

**Fig 6:**
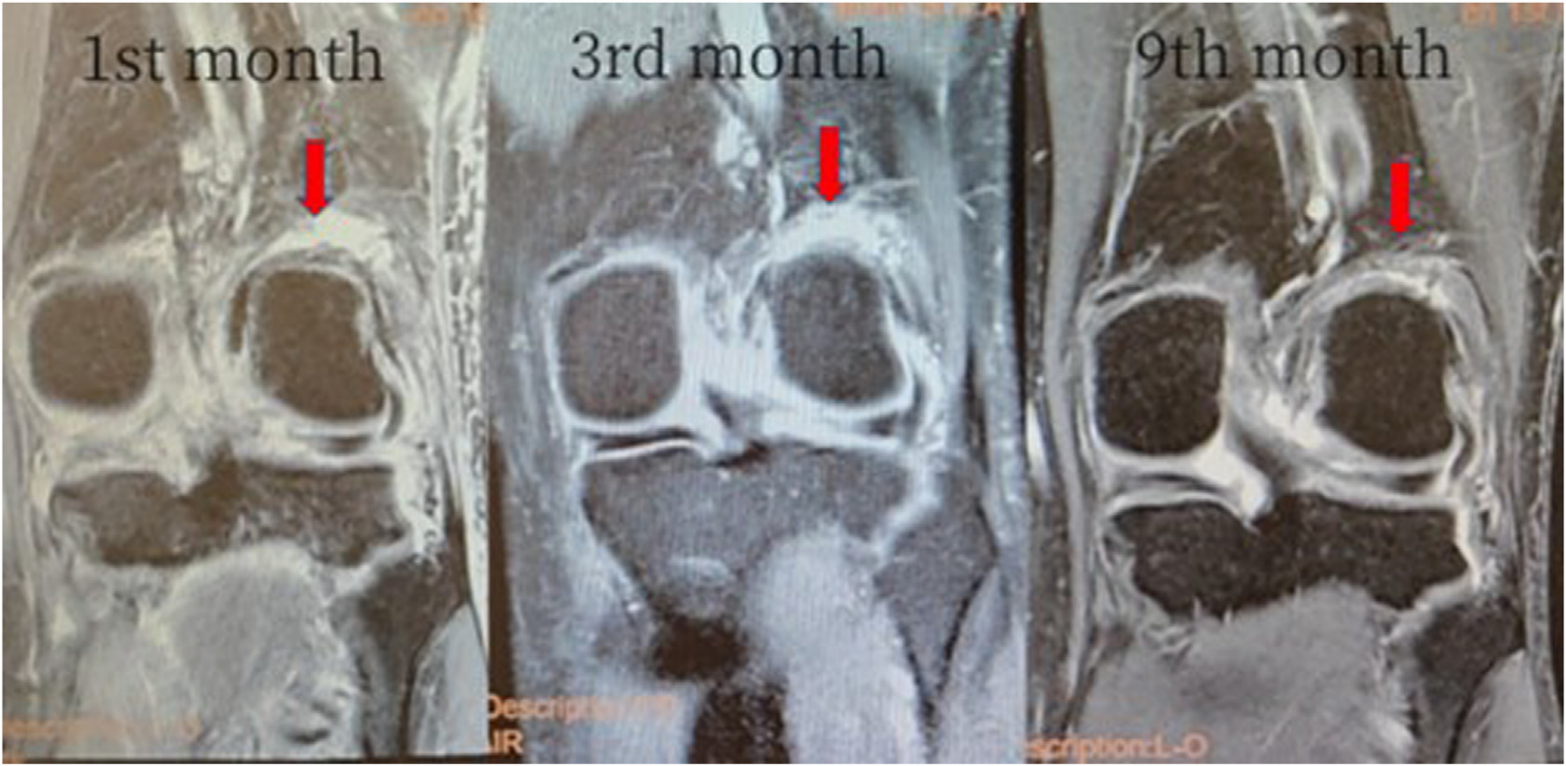
Serial coronal T2-weighted MRI of a 35-year-old male patient after left knee OTT + anterior lateral ligament combined reconstruction surgery acquired 1, 3, and 9 months postoperatively. The arrow shows that the boundary between the transplanted tendon in the OTT part and the surrounding tissues has become gradually blurred. (MRI, magnetic resonance imaging; OTT, over-the-top.)

However, compared with isolated ACLR, the combined intra-articular and extra-articular surgical operation is more complex and more invasive because extra-articular reconstruction requires larger skin incisions and dissection to complete.^12^ The surgical method we describe has the following characteristics. (1) The process of the intra-articular ACLR and femoral OTT technique is completed under the arthroscope, the reconstruction of the ALL outside the joint is completed using a percutaneous technique, and the entire surgical process is minimally invasive (Fig 5). (2) The double anchor nails located inside and outside the joint suspend and fix the graft over the femoral condyle, ensuring that the graft segment on the femoral condyle has relative stability during knee joint activities, avoiding additional femoral tunnels and making revision simpler. (3) Under arthroscopic monitoring, we have created a soft-tissue channel between the posterior joint capsule and the posterior femoral condyle for passage of the graft, which provides the possibility of healing between the graft and the joint capsule soft tissue. In the follow-up, it was observed on magnetic resonance imaging that the boundary between the graft at the OTT position and the joint capsule gradually became blurred (Fig 6), which is the result of soft-tissue interface healing. We believe that this way of soft-tissue healing is easier to complete than the healing between tendon and bone. (4) Using the same graft for the integrated reconstruction of ACL and ALL can provide multidirectional stability, effectively eliminate pivot shift, effectively enhance the strength of the reconstructed ligament, and conform to the concept of early rehabilitation. Its minimally invasive and improved rotational stabilization effectiveness is indicated in ACRL revision surgery. The limitations of the technique include that it is time-consuming, slightly cumbersome, and only short follow-up is available in patients thus far (Table 2).

**Table 2:** Advantages and Disadvantages

Advantages	Disadvantages
No more need of femur tunnel during reconstruction, making the process more straightforward.	Longer operation time compared with traditional procedures.
Over-the-top grafts and soft-tissue healing may be easier than healing at the tendon-bone interface	Slightly more complex compared with conventional methods.
Provides stability in multiple directions within the joint, allowing for earlier functional rehabilitation.	Needing more follow-up period

## Disclosures

The authors (G.Y., S.H.) declare that they have no known competing financial interests or personal relationships that could have appeared to influence the work reported in this paper.

## Supplementary Data

Video 1 The arthroscopic operation process of the over-the-top reconstruction of the anterior cruciate ligament with integration inside the joint.
